# Visual encoding and fixation target selection in free viewing: presaccadic brain potentials

**DOI:** 10.3389/fnsys.2013.00026

**Published:** 2013-06-27

**Authors:** Andrey R. Nikolaev, Peter Jurica, Chie Nakatani, Gijs Plomp, Cees van Leeuwen

**Affiliations:** ^1^Laboratory for Perceptual Dynamics, University of LeuvenLeuven, Belgium; ^2^Laboratory for Advanced Brain Signal Processing, RIKEN Brain Science InstituteWako-shi, Japan; ^3^Functional Brain Mapping Laboratory, Université de GenèveGenève, Switzerland

**Keywords:** saccades, EEG, presaccadic interval, attention, visual encoding, saccade guidance, change detection, heat maps

## Abstract

In scrutinizing a scene, the eyes alternate between fixations and saccades. During a fixation, two component processes can be distinguished: visual encoding and selection of the next fixation target. We aimed to distinguish the neural correlates of these processes in the electrical brain activity prior to a saccade onset. Participants viewed color photographs of natural scenes, in preparation for a change detection task. Then, for each participant and each scene we computed an image heat map, with temperature representing the duration and density of fixations. The temperature difference between the start and end points of saccades was taken as a measure of the expected task-relevance of the information concentrated in specific regions of a scene. Visual encoding was evaluated according to whether subsequent change was correctly detected. Saccades with larger temperature difference were more likely to be followed by correct detection than ones with smaller temperature differences. The amplitude of presaccadic activity over anterior brain areas was larger for correct detection than for detection failure. This difference was observed for short “scrutinizing” but not for long “explorative” saccades, suggesting that presaccadic activity reflects top-down saccade guidance. Thus, successful encoding requires local scanning of scene regions which are expected to be task-relevant. Next, we evaluated fixation target selection. Saccades “moving up” in temperature were preceded by presaccadic activity of higher amplitude than those “moving down”. This finding suggests that presaccadic activity reflects attention deployed to the following fixation location. Our findings illustrate how presaccadic activity can elucidate concurrent brain processes related to the immediate goal of planning the next saccade and the larger-scale goal of constructing a robust representation of the visual scene.

## Introduction

While scrutinizing a visual scene, observers typically make saccadic eye movements from one fixation location to the next. During fixation intervals, two component processes can be distinguished. Visual encoding, the first of these processes, serves the overall goal of building a robust representation of the scene. Visual information extracted from attended and fixated locations is accumulated across eye movements, (Melcher, 2001, 2006; Tatler et al., 2003, 2005; Pertzov et al., 2009) and is transferred to visual short- and long-term memory (Hollingworth and Henderson, 2002; Henderson and Hollingworth, 2003; reviewed in Hollingworth, 2004).

The second component process serves a more immediate goal of perception: deciding where to move the eyes next. Selection of the next fixation target involves directing covert attention, which precedes the execution of a saccade to the next target (Hoffman and Subramaniam, 1995; Deubel and Schneider, 1996). The selection is controlled by bottom-up target salience, in combination with top-down relevance of the target (reviewed in Awh et al., 2006).

Visual encoding and next-target selection are likely to share informational resources: information accumulated during the current fixation involves the spatial and semantic properties of a scene that determine what would be an interesting target for the next fixation. We may therefore expect that both encoding and next-target selection draw on the same attentional mechanisms, and that their neural markers overlap in time.

The goal of this study is to pinpoint and analyze in scalp-recorded electrical brain activity the processes of visual encoding and target selection as they evolve during the fixation interval. Our analysis is focused on the interval preceding saccade onsets. This interval has been studied in relation to covert attention shifts to the next fixation target, the initial phase of trans-saccadic remapping of receptive fields, and oculomotor preparation (reviewed in Melcher and Colby, 2008; Mathot and Theeuwes, 2011). Correspondingly, scalp-recorded electrical brain activity in the presaccadic interval reflects directing spatial attention (Wauschkuhn et al., 1998; Krebs et al., 2012), trans-saccadic remapping (Parks and Corballis, 2008), and oculomotor preparation (Kurtzberg and Vaughan, 1982; Csibra et al., 1997; Richards, 2003).

The results of these studies throw light on brain processes related to the control of eye movements. Little is known, however, about the presaccadic activity related to the accumulation of visual information during viewing a scene. In previous work we found that this activity is predictive of performance in a change-detection task (Nikolaev et al., 2011). Since change detection depends on successful accumulation of scene information (Simons and Rensink, 2005), we can use this as a criterion to study encoding in the presaccadic interval.

We may expect effects of encoding during the presaccadic interval to be modulated by periodic systematic tendencies in scene viewing (Tatler and Vincent, 2008), in other words, by viewing strategies. Viewing strategies are reflected in characteristic sequences of saccades and fixations (Unema et al., 2005; Tatler and Vincent, 2008; Graupner et al., 2011; Mills et al., 2011). For example, global scanning is reflected in large-amplitude saccades and short fixation durations, whereas local scanning is reflected in small-amplitude saccades and long fixation durations (Unema et al., 2005; Tatler and Vincent, 2008). Although patterns of short and long saccades tend to alternate throughout free viewing episodes (Tatler and Vincent, 2008; Mills et al., 2011), long saccades predominate during the first 2 s of free viewing (Unema et al., 2005; Pannasch et al., 2008; Graupner et al., 2011). This suggests that in the course of scrutinizing a scene a shift from global to local scanning strategy occurs. We may posit a corresponding shift from bottom-up to top-down saccade guidance (Findlay and Walker, 1999). Following these previous studies, we will analyze presaccadic potentials related to short, medium and long saccades separately, in order to determine scanning strategy and its influence on encoding.

Saccade size is reflected in eye fixation-related potentials (EFRPs) time-locked to the fixation onset. Graupner et al. (2011) occasionally presented circular distractors at fixation location, in 100 ms after the fixation onset. The amplitude of the distractor-evoked EFRP components depended on size of the preceding and/or following saccades. In contrast with Graupner et al. (2011), our study considers viewing strategies as they are reflected in the electrical brain activity *before* saccade onset.

The scalp-recorded activity in the presaccadic interval is characterized by a slow positive wave over parietal brain areas, which is called the antecedent potential (Becker et al., 1973; Kurtzberg and Vaughan, 1982; Moster and Goldberg, 1990; Csibra et al., 1997; Richards, 2003; Parks and Corballis, 2008), as well as by positive potentials over frontal areas (Richards, 2000; Gutteling et al., 2010).

The parietal and frontal potentials may reflect activity of, respectively, the lateral intraparietal area (LIP) and the frontal eye field (FEF). On the one hand, these areas are strongly interconnected (Andersen et al., 1985; Bullier et al., 1996) and share eye movement control functions between them (Medendorp et al., 2011): they map salient or task-relevant objects (Gottlieb and Balan, 2010) and are involved in guidance of spatial attention (Thompson et al., 1996; Goldberg et al., 2006). On the other hand, these areas are functionally distinct. For example, the frontal area is more closely associated with oculomotor functions than the parietal area (Curtis and D'Esposito, 2006; Connolly et al., 2007); in addition, whereas in top-down attention tasks frontal neurons respond earlier than parietal ones to saccade target location, in bottom-up attention tasks it is the other way around (Buschman and Miller, 2007). We will therefore distinguish the presaccadic activity over frontal and parietal areas in our analyses.

In sum, in our study we will distinguish between processes of visual encoding and target selection, as they are reflected in the electrical brain activity in the presaccadic interval. Since these processes may differ in bottom-up and top-down scanning strategies associated with saccade size, we will consider the presaccadic activity for different saccade sizes separately.

## Materials and methods

We re-analyzed data from a previously published study. The details of the experimental procedure can be found elsewhere (Nikolaev et al., 2011). Here we outline the main steps only.

### Participants

Nineteen healthy participants (ages 19–24, median age 20, 5 men) took part in the study. The main analyses were done in seventeen participants who had sufficient numbers of epochs per condition, as described below. All participants gave written informed consent. The study was approved by Institutional Review Board No.2 (Research Ethics Committee) of RIKEN Brain Science Institute (Wako-shi, Japan) where we conducted the experiment.

### Stimuli

We used 48 pairs of color photo images of real-world scenes from the study by Rensink et al. (1997). The images were 28° wide and 22° high. There were three types of differences between images in a pair: color, position, or presence/absence of an object. Color difference involved either an object or part of the background. Position difference referred to displacement of an object by several degrees of visual angle. Presence/absence involved the occurrence or non-occurrence of an object in the display. Stimuli were presented using custom-made software written in Python using Vision Egg interface (Straw, 2008).

### Procedure

Stimuli were presented on a 21-in. CRT Gateway monitor placed at 85 cm from the participant in a dimly lit room. In a practice session participants were familiarized with examples of three types of difference between images. In the main session, after stable fixation on a central crossways reached, the first image of a pair, the *memorization display*, was presented for 20 s. Then, after a 1-s mask, the second image, the *search display*, was presented until response but no longer than 20 s. We asked participants to memorize the first image and detect a change in the second image. Participants had to respond with two mouse clicks: first, as soon as a change was detected, second, after placing the cursor on the change region. Feedback was immediately given by showing the image with a red ellipse over the changed region. The order of images was counterbalanced across participants, such that half of the time each image was used as a memorization or search display.

### Eye movement recording

Eye movements were recorded with a video-based infrared eye-tracking system (EyeLink 1000/Tower, SR Research Ltd., Ontario, Canada). The participant's head was stabilized using a chin and forehead rest. The right eye was tracked with a sampling rate of 500 Hz. The eye-tracker was calibrated using nine points: in the center, four corners and mid-points of the four sides of the screen. Mean difference between calibration and validation measurement was kept below 1.5°.

### EEG recording

EEG was recorded with a Nihon Kohden MEG-6116 amplifier using an ECI electrode cap (Electro-Cap International, Inc., Eaton, USA) with 15 electrodes (F3, F4, C3, C4, P3, P4, O1, O2, T3, T4, T5, T6, Fz, Cz, Pz) placed according to the international 10/20 system with electrode FCz as ground and the linked mastoids as reference. Data were recorded with 0.5 Hz high-pass and 100 Hz low-pass online filters (and a 50 Hz notch filter) and digitized at 500 Hz.

The analog output of the EyeLink system was connected to the EEG amplifier and the eye movement horizontal (X) coordinate was recorded as an additional channel (the *EyeLink channel*) along with the regular EEG channels. To mark the eye movement events in the EEG data, we translated the time stamps of the events from the EyeLink data file. To that end, we correlated the EyeLink channel (recorded by the EEG acquisition computer) and the X coordinate time-series from the EyeLink data file (recorded by the eye movement acquisition computer). In each trial (which consisted of a presentation of two displays, a memorization and a search display), we selected a blink-free segment and determined the lag of maximum correlation between the EyeLink channel and the time-series from the EyeLink data file using normalized cross-correlation. The correlation coefficient for synchronized segments was never below 0.98. Finally, the time stamps of the eye movement events (computed by the EyeLink system) were adjusted for this lag and inserted in the EEG data as markers of saccades and fixations.

### Eye movement analysis

We analyzed eye movements and EEG from the 20-s presentations of the memorization display.

Onset time, location, and duration of saccades and fixations were determined by EyeLink software. In our eye movement and EEG analyses we defined a fixation-related epoch by the combination of parameters of a fixation and a following saccade: the fixation has to be longer than 200 ms and shorter than 2000 ms, and the following saccade has to be shorter than 60 ms. Presaccadic intervals containing blinks were excluded.

### Fixation heat maps

**Figure 1:**
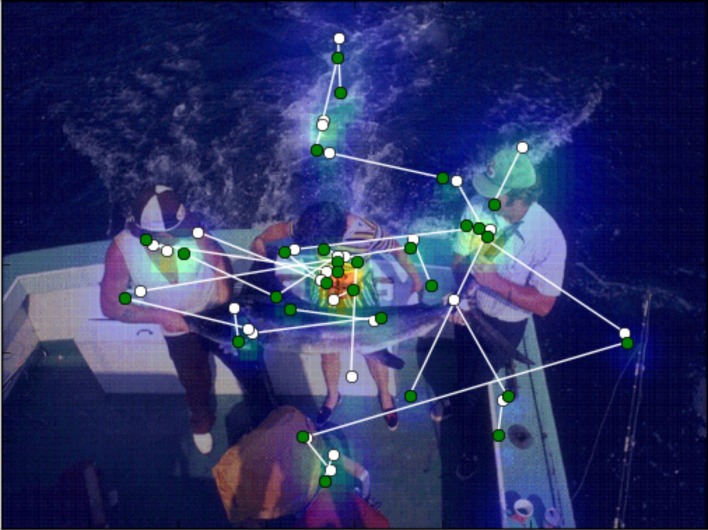
**Temperature map computed as a function of fixation duration and fixation density.** The saccades are superimposed on the image. Green circles designate the starting points of saccades and white circles designate the end points. The direction of the saccade is given a positive or negative sign, depending on the difference between the temperature values of start and end points: positive if the end temperature is higher than the start, negative if vice versa.

For each image and each individual participant, heat maps were computed as a function of fixation duration and fixation density (Figure 1). All fixations collected during presentation of a memorization display were accumulated to compute a heat map using visual kernel density estimation (Jones et al., 1996). Contribution of each fixation was represented by a Gaussian kernel with unit amplitude and spread proportional to the fixation duration

$$t_{i(X,Y)}=\text{exp }\left[-0.5\left(\frac{{\left(X-x_{i}\right)}^{2}+{\left(Y-y_{i}\right)}^{2}}{\sigma^{2}}\right)\right]$$

where *t*_i_ is the temperature contribution of the *i*th fixation, (*X, Y*) is the pixel coordinate within the stimulus image, (*x_i_, y_i_*) is the center of *i*th fixation, and spread (σ = τ*_i_/k*) is proportional to the duration (τ*_i_*) of the fixation. The linear bandwidth parameter *k* = 20 was determined visually to obtain a smooth unimodal density function above each cluster of fixations. A larger bandwidth covers a wider area and reduces the relative advantage of pixels in near proximity of the center of the fixation. Longer fixations allow more time and space for exploration of the surroundings of the center of fixation using micro-saccades. The final heat map was the average of all contributions *T* = sum*_i_t_i_*.

We superimposed for each saccade the start and end points on the images (Figure 1) and extracted their temperatures as the average of all temperatures located within a radius of 0.5° of visual angle. The radius was chosen in accordance with the measurement error in the eye-tracking equipment.

High temperature on the heat maps indicates areas with long fixation durations and small saccade sizes, i.e., regions which were carefully scrutinized using a local scanning strategy. Low temperature areas, by contrast, indicate regions that were only occasionally visited during global scanning or not visited at all.

As an indicator of target selection for the next fixation we used the difference in temperature between the start and end point of a saccade. The difference can be understood as a contrast in anticipated task-relevant information between locations. We considered a saccade as going in the *positive* direction if the temperature at the end point was higher than at the start point, and vice versa if the saccade direction was *negative*.

### Division in saccade size bins

Since we proposed that the corresponding processes depend on viewing strategies as reflected in saccade size, we divided up the data into three bins according to saccade size: short, medium and long. Three bins were used in order to secure a minimal number of epochs per condition (which was set to 50) for a sufficiently large number of participants. We still had to exclude two participants who did not have enough epochs in one of the conditions. Thus, eye movement and EEG analyses were performed in 17 participants.

### Eye fixation-related potential analysis

For the EEG analysis we used Brain Vision Analyzer software (Brain Products GmbH, Gilching, Germany). The EEG signal was filtered with a Butterworth zero-phase filter with a high cut-off frequency of 30 Hz, 12 dB/oct. From the 15 recorded channels, two temporal channels (T3 and T4) were excluded because of artifacts resulting from muscle activity.

We analyzed EEG in the fixation intervals preceding saccade onsets. We used only those EEG epochs that corresponded to fixation intervals without blinks. These epochs are by definition free of artifacts caused by eyelid and eyeball movements (Dimigen et al., 2011). However, if a blink had occurred in the preceding fixation interval or if the fixation was short, the tail of the activity evoked by the blink or the saccade would still have had a chance to contaminate the current fixation interval. We used ICA to remove these effects from the EEG data (Jung et al., 2000) as follows. We selected a 300-s interval from 100 to 400 s of continuous EEG recording, which included a number of blinks and saccades. The data from 13 regular EEG channels in this interval constituted the training dataset for computing the unmixing matrix. Then in each participant we identified the ICA components which picked up eye movement artifacts. The time course of these components mirrored blinks or saccades in the “EyeLink channel” recorded together with EEG channels. These components had typical topography with a maximum at the frontal (F3, Fz, F4) sites: the blink-related component had a symmetrical maximum at the frontal electrodes; the saccade-related component had a characteristic asymmetrical topography over the frontal sites reflecting the directionality of the saccades. Finally, the whole duration of the EEG was reconstructed without these components. As expected, ICA artifact correction primarily affected the activity at the frontal sites. Since the unmixing matrix was computed using an EEG interval encompassing all experimental conditions, it is unlikely that ICA selectively or systematically altered the presaccadic activity in a certain condition.

The markers of saccade onset were computed by EyeLink software and were incorporated into the EEG time series. EEG was segmented into epochs from −200 ms to 50 ms relative to saccade onset. Using a semi-automatic artifact rejection procedure, we excluded epochs if the absolute voltage difference exceeded 50 μV between two neighboring sampling points and if the amplitude exceeded +100 or −100 μV. After the artifact rejection the mean number of epochs per condition was 158 (SD: 36; range 109–220).

For each participant, we set the number of epochs to be equal in all conditions. To that end, we first identified the condition where the number of epochs was minimal. Then for all other conditions we randomly selected a number of epochs equal to this minimum.

Based on visual inspection of the grand averaged potentials (Figure 4A), we selected the interval −100 to 20 ms before the saccade onset for statistical evaluation of the presaccadic activity. We computed the mean amplitude of this interval. In addition, we evaluated the peak-to-peak amplitude of the saccadic spike potential by measuring the difference between the positive and negative peaks of this potential.

We averaged the epochs for each participant and condition separately. As we discussed in the Introduction, one of our goals was to distinguish between activities over the frontal and parietal brain areas. Therefore we preselected anterior (F3, F4, Fz, C3, C4) and posterior (P3, P4, Pz, O1, O2) groups of electrodes and averaged the potentials across electrodes within each group.

For the baseline correction we selected a 20-ms interval in the beginning of the presaccadic epoch (i.e., −200 to 180 ms before the saccade onset).

### Statistical analysis

We considered three main factors: Saccade size (short, medium, long), Correctness (Correct detection vs. failure) and Saccade direction (positive vs. negative). For the analysis of the eye movement measures, unless otherwise stated, we used univariate repeated-measures ANOVA with these factors. As for the event-related potentials (ERP) analysis, the effect of scalp locations on amplitude differences is not an additive effect but a multiplicative one; therefore additive ANOVA models cannot unambiguously evaluate topographic differences (McCarthy and Wood, 1985). For this reason, we treated amplitude in the anterior and posterior electrode groups as two dependent variables in a multivariate design (MANOVA). Whenever MANOVA revealed a significant effect, we proceeded to univariate ANOVA follow-up analyses and *post-hoc* tests, in order to identify the specific dependent variables that contributed to the effect. In the univariate ANOVA we applied the Huynh-Feldt correction (ε) of *p*-values associated with more than two degrees of freedom, in order to compensate for violation of sphericity. For *post-hoc* analyses we used Fisher's LSD (Least Significant Difference) test.

For presaccadic activity, the correlation between anterior and posterior signals was *r*_(17)_ = 0.35. This is in accordance with the MANOVA requirement that the dependent variables should be moderately correlated with each other (i.e., 0.20–0.60; Meyers et al., 2006). The mean peak-to-peak amplitude of the saccadic spike potential, however, highly correlated between the anterior and posterior electrode groups [*r*_(17)_ = 0.97]. We therefore ran two separate ANOVAs on the anterior and posterior saccadic spike potentials.

## Results

### Eye movement results

**Figure 2:**
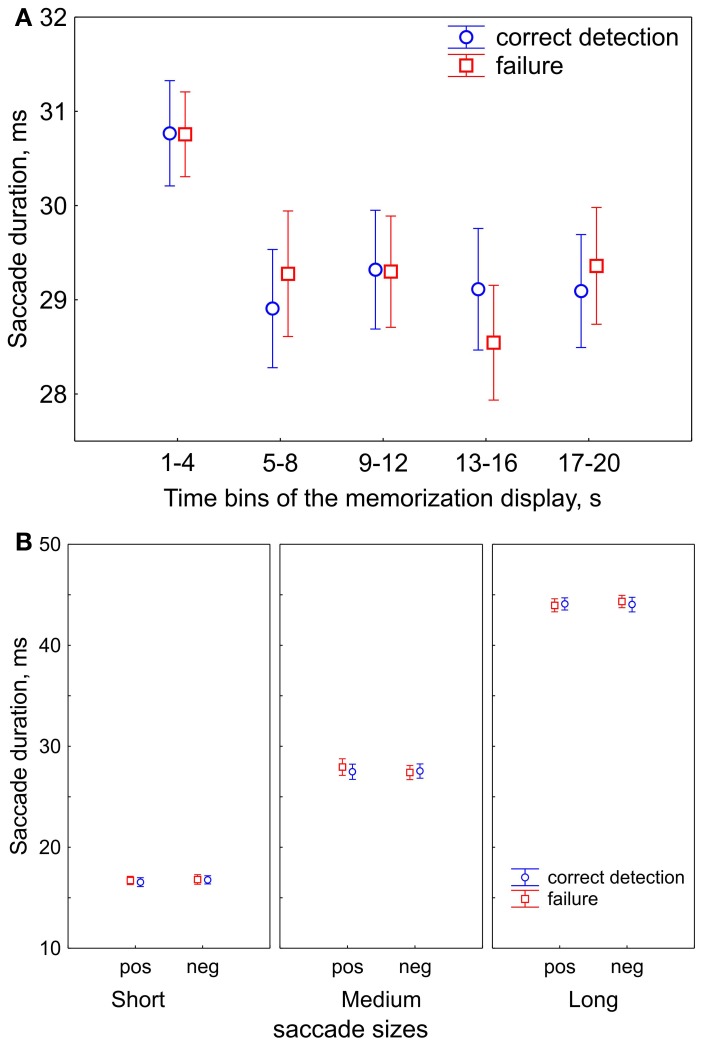
**Saccade durations (sizes). (A)** Saccade durations in the course of free viewing during 20-s presentations of the memorization display for subsequent correct detection and failure. The 20-s presentation of the memorization display was divided in five 4-s time bins. Saccade durations decrease after the first bin. **(B)** The ranges of saccade durations after division in 3 saccade size bins: short, medium, and long saccades. Saccade sizes are shown for positive (“pos”) and negative (“neg”) saccade directions, and for correct detection and failure, in order to demonstrate that their values did not differ between conditions. The data points are the means and the error bars represent standard errors across 17 participants.

First, we tested for changes in viewing strategy. We divided 20-s memorization intervals into five 4-s time bins. In each bin we computed the saccade duration for correct detection and failure. We found that saccade duration decreased after the first bin and then remained unchanged (Figure 2A). A repeated-measures ANOVA with factors of Time Bins (5 levels) and Correctness (correct detection vs. failure) revealed an effect of Time Bins [*F*_(4, 64)_ = 8.9, *p* < 0.001, Huynh-Feldt ε = 0.82] and no correctness effect nor interaction. *Post-hoc* test showed that the effect of Time Bins occurred because of longer saccades in the first bin than all other ones (all *p* < 0.001). This finding can be understood as a shift in emphasis from global to local viewing strategy in the course of free viewing, consistently with previous reports (Unema et al., 2005; Pannasch et al., 2008; Graupner et al., 2011).

The shift in viewing strategy may affect the processes of visual encoding and fixation target selection. Since we determined strategy according to saccade size (as reflected in saccade duration), we divided up the data into three bins accordingly: short, medium and long. Figure 2B illustrates the ranges of saccade durations after binning. A repeated-measures ANOVA with factors of Saccade size (short, medium, long), Saccade direction (positive vs. negative), Correctness (correct detection vs. failure) revealed that the saccade durations did not differ between negative and positive saccade directions [*F*_(1, 16)_ = 0.09, *p* = 0.77] and between correct detection and failure [*F*_(1, 16)_ = 1.7, *p* = 0.22]. This suggests that the oculomotor component of saccade preparation was similar for all conditions within a saccade size bin.

**Figure 3:**
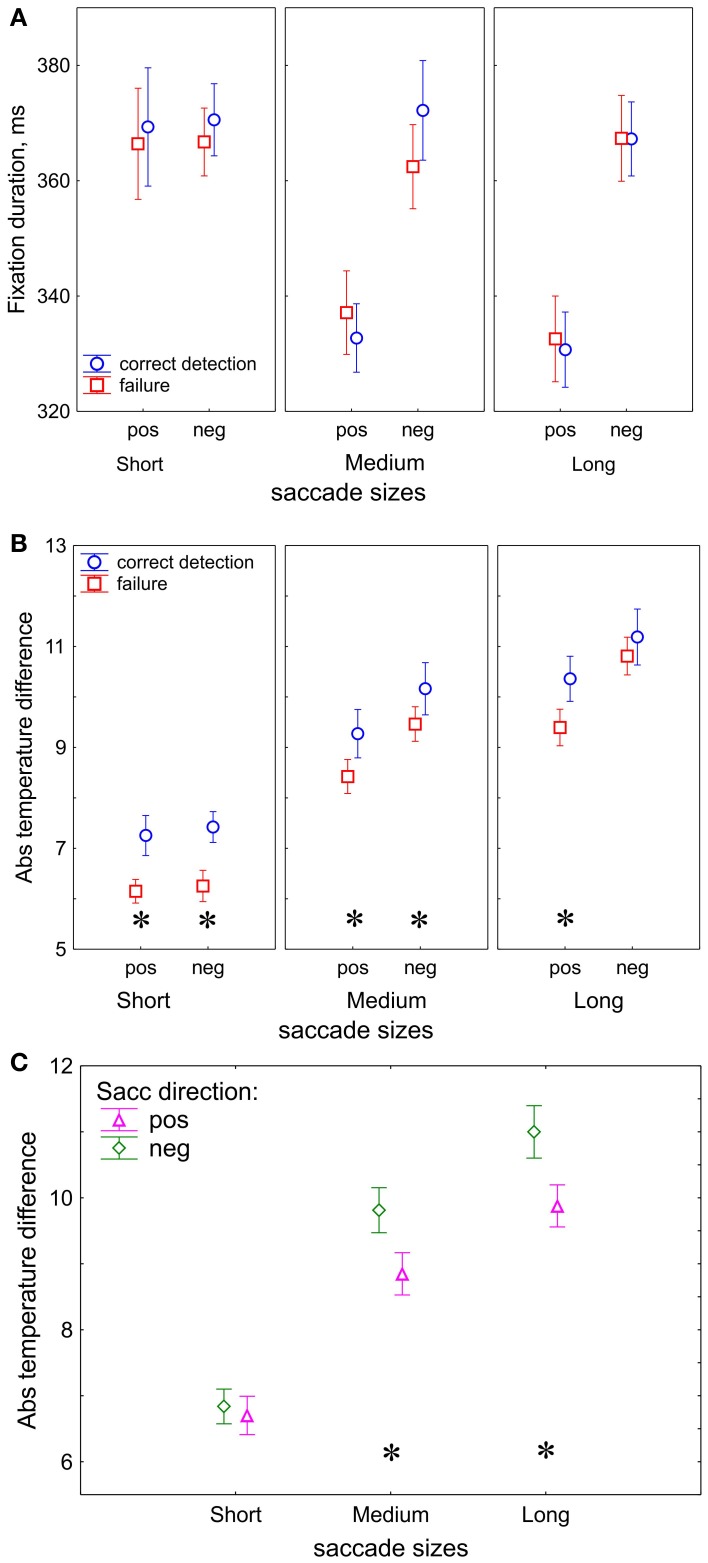
**Eye movement results. (A)** Duration of the fixations preceding the saccades used for dividing trials into saccade size bins. **(B)** Absolute temperature difference on the fixation heat maps, correctness effect. **(C)** Absolute temperature difference, saccade direction effect. “pos” indicates positive and “neg” indicates negative saccade direction. The data points are the means and the error bars represent standard errors across 17 participants. The asterisks designate significant differences between correct detection and failure.

For the three saccade sizes, we considered the duration of their *preceding* fixations (Figure 3A). Saccade size conditions showed a prominent effect on preceding fixation duration [*F*_(2, 32)_ = 10.7, *p* < 0.001, Huynh-Feldt ε = 1.0] and there was an interaction between Saccade size and Saccade direction [*F*_(2, 32)_ = 37.1, *p* < 0.001, Huynh-Feldt ε = 1.0]. These effects indicate shorter fixation durations for positive than for negative direction for medium (*p* < 0.001) and long (*p* < 0.001) saccades. Correctness did not yield an effect [*F*_(1, 16)_ = 0.4, *p* = 0.52]; no further interactions were found.

In contrast to fixation duration, the *temperature difference* between fixation locations appears to be sensitive to correctness of change detection (Figure 3B). The absolute values of the temperature difference was higher in correct detection than in failure [*F*_(1, 16)_ = 5.7, *p* = 0.03].

In our study we used the temperature difference as an indicator of fixation target selection since it represents the contrast of task-relevant information between two fixation locations. The absolute temperature difference was larger for positive than negative direction [*F*_(1, 16)_ = 33.7, *p* < 0.001]. In order to evaluate how the target selection depends on saccade sizes we compare the absolute temperature differences for short, medium and long saccades. After a short saccade the gaze is likely to land into an image region with similar temperature, whereas after a long saccade the gaze may land into a region with a different temperature. As expected, we found a prominent increase of the temperature difference with saccade size [*F*_(2, 32)_ = 93, *p* < 0.001, Huynh-Feldt ε = 1.0].

In addition, the processes of target selection are different for the short and long saccades. We observed an interaction between Saccade size and Saccade direction: *F*_(2, 32)_ = 4.98, *p* = 0.03, Huynh-Feldt ε = 0.70) which was occurred because of the larger temperature difference in the negative than positive direction for the long (*p* < 0.001) and medium (*p* < 0.001) saccades, but not for short (*p* = 0.57) ones (Figure 3C).

### EEG results

**Figure 4:**
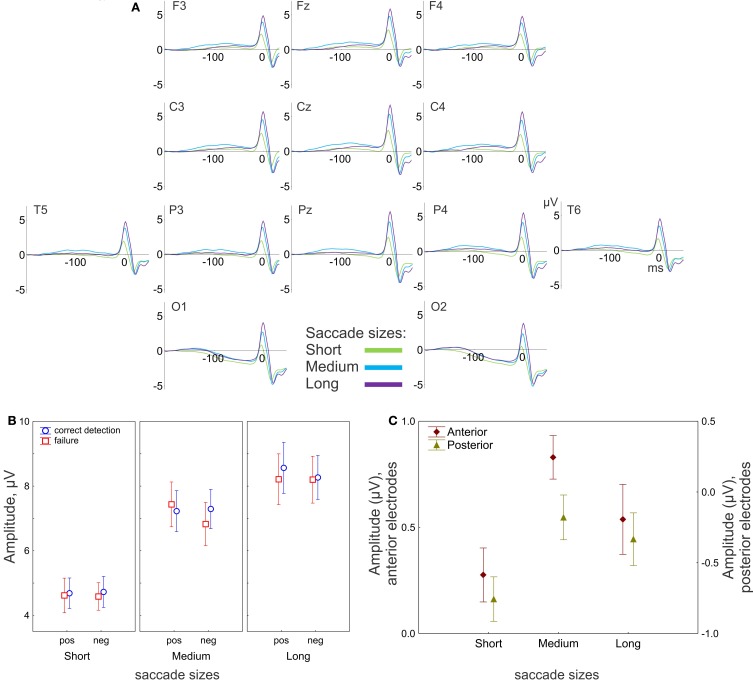
**EEG results: effect of saccade size. (A)** Grand-averaged potentials for 13 electrodes for three saccade sizes. 0 ms is the saccade onset. **(B)** The peak-to-peak amplitude of the saccadic spike potential. The amplitude gradually increases with saccade size. **(C)** Amplitude of the presaccadic activity (the mean in the interval −100 to 20 ms prior to the saccade), effect of saccade size for anterior (F3, F4, Fz, C3, C4) and posterior (P3, P4, Pz, O1, O2) electrode group. “pos” indicates positive and “neg” indicates negative saccade direction. The data points are the means and the error bars represent standard errors across 17 participants.

Figure 4A shows the scalp topography of the spike potentials averaged over 17 participants relative to saccade onset for three saccade sizes.

#### Saccadic spike potential

**Figure A1:**
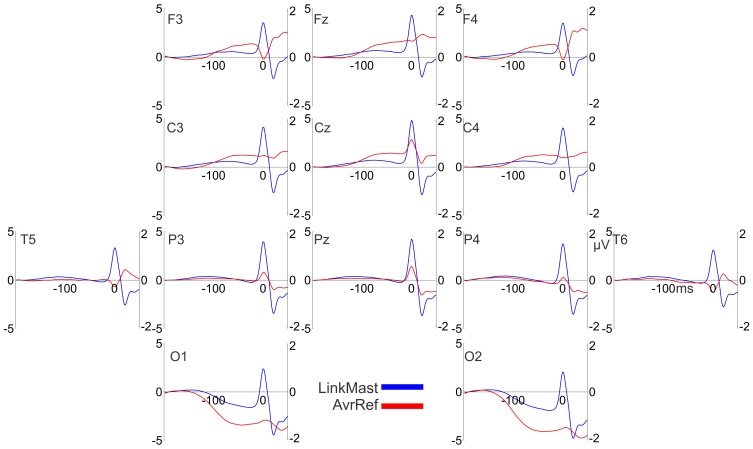
**The effect of reference on the polarity of the saccadic spike potentials.** Grand-averaged potentials (17 participants, averaged across all conditions) for 13 electrodes relative to the linked mastoid (“LinkMast” used in the paper, the left Y axis) and the average reference (“AvrRef”, the right Y axis). 0 ms is the saccade onset. The average reference inverts the polarity of the saccadic spike potentials over the frontal sites.

The saccadic spike potentials emerged as a biphasic wave of the same positive polarity in all recording sites. Typically the positive polarity is observed only in the parietal sites and alternates between positive and negative polarities in the frontal sites depending on saccade direction (Csibra et al., 1997). The observed topographical distribution of the polarity was a consequence of the linked-mastoid reference used: re-referencing to the average reference inverted the polarity of the saccadic potentials over the frontal sites, keeping the positive polarity over the parietal sites, as it is illustrated in Figure A1 in the Appendix (for a similar effect see Figure 6B in Plochl et al., 2012). However, even for the mastoid reference used, the peak-to-peak amplitude of the saccadic potential was much larger in the posterior than in the anterior electrode group [7.9 μV (SEM 0.52) and 6.7 μV (SEM 0.59), respectively, *t*_(17)_ = 7.8, *p* < 0.001], consistently with a parietal amplitude maximum of the saccadic potential (Csibra et al., 1997; Keren et al., 2010).

The amplitude of the saccadic potential strongly depends on saccade size: the amplitude linearly increased with saccade size for the anterior [*F*_(2, 32)_ = 72, *p* < 0.001, ε = 0.64] and posterior [*F*_(2, 32)_ = 81, *p* < 0.001, ε = 0.7] electrode groups (Figure 4B). There were no other effects, nor interactions, so the saccadic potential did not differ between conditions in any of the preselected saccade size (short, medium or long). This result is consistent with the common finding that the saccadic spike potential reflects only saccade sizes and is not sensitive to cognitive influences (e.g., Keren et al., 2010).

#### Presaccadic activity

The presaccadic activity is represented by a slow positive wave with a maximum about 100 ms before saccade onset (with the exception of O1 and O2 sites). The amplitude of the presaccadic activity (−100 to 20 ms before the saccade) averaged across all conditions appeared much larger in the anterior than in the posterior electrode group (Figure 4C).

#### Effects of saccade size

The MANOVA revealed a significant effect of saccade size on the amplitude of presaccadic activity [*F*_(4, 13)_ = 9.9, *p* < 0.001; Wilk's Λ = 0.25]. The effect was significant for both anterior [univariate *F*_(2, 32)_ = 7.9, *p* = 0.002, ε = 1.0] and posterior [univariate *F*_(2, 32)_ = 7.0, *p* = 0.003, ε = 1.0] electrode groups. Figure 4C shows smaller amplitude for short than for medium saccades for both electrode groups (both *post-hoc p* < 0.001). For the long saccades the amplitude was smaller than for the medium saccades, for the anterior (*p* = 0.04) but not for the posterior electrode group (*p* = 0.34).

#### Correctness effect

**Figure 5:**
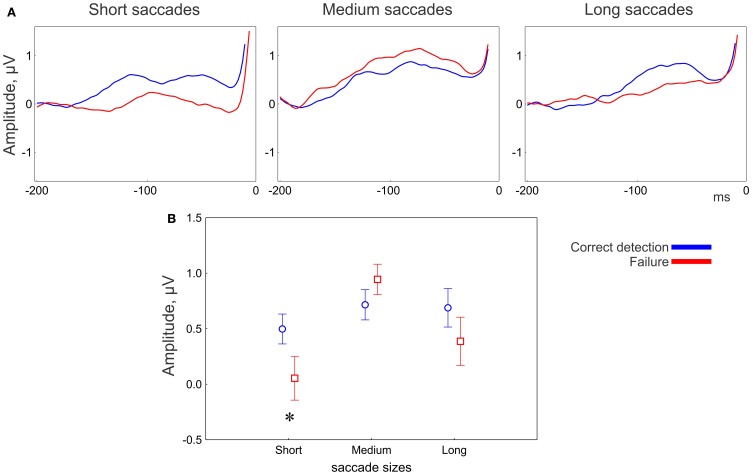
**EEG results: effect of correctness for the anterior group of electrodes. (A)** Grand averaged potentials for three saccade sizes. 0 ms is saccade onset. **(B)** Amplitude of the presaccadic activity (the mean in the interval −100 to 20 ms prior to the saccade) for correctness conditions for 3 saccade sizes. The asterisk designates a significant difference between correct detection and failure. The data points are the means and the error bars are the standard errors across 17 participants.

The main effect of correctness of change detection on the presaccadic amplitude was not significant; however, the correctness depended on saccade size and electrode location (Figure 5A). The MANOVA revealed an interaction between Correctness and Saccade size [*F*_(4, 13)_ = 3.2, *p* = 0.048; Wilk's Λ = 0.50]. The univariate ANOVAs revealed an interaction tendency [*F*_(2, 32)_ = 2.7, *p* = 0.097, ε = 0.84] only for the anterior group, with larger amplitude for correct detection than for failure, for short (*post-hoc p* = 0.049) but not for medium (*p* = 0.3) or long (*p* = 0.18) saccades (Figure 5B).

#### Saccade direction effect

**Figure 6:**
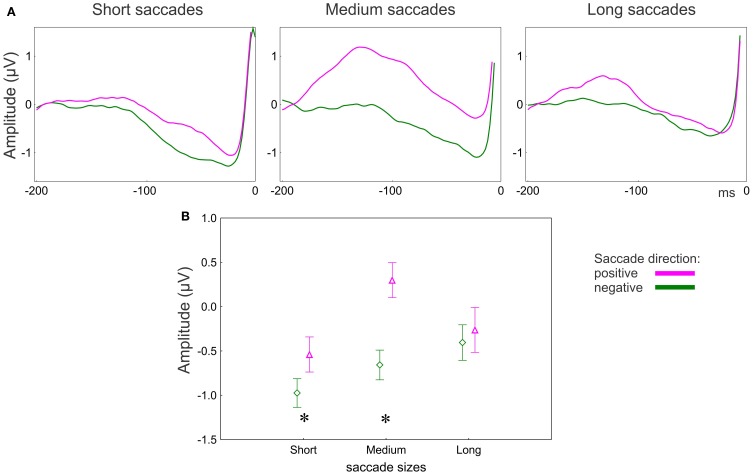
**EEG results: effect of saccade direction for posterior group of electrodes. (A)** Grand averaged potentials for three saccade sizes for the posterior group of electrodes. 0 ms is saccade onset. **(B)** Amplitude of the presaccadic activity (the mean in the interval −100 to 20 ms prior to the saccade) for 3 saccade sizes. Asterisks designate significant differences between negative and positive saccade directions. The data points are the means and the error bars represent standard errors across 17 participants.

The presaccadic amplitude appeared larger for saccades in the positive than in the negative saccade direction (Figure 6). The MANOVA revealed an effect of Saccade direction [*F*_(2, 15)_ = 8.3, *p* = 0.004; Wilk's Λ = 0.48]. The effect of Saccade direction was significant for both anterior [univariate *F*_(1, 16)_ = 11.6, *p* = 0.003] and posterior [univariate *F*_(1, 16)_ = 15.3, *p* = 0.001] electrode groups.

The effect of Saccade direction on the presaccadic amplitude depends on saccade size and electrode location (Figure 6A). The univariate ANOVA revealed an interaction between Saccade direction and Saccade size [*F*_(2, 32)_ = 4.0, *p* = 0.03, ε = 1.0] for the posterior electrode group only. The amplitude was larger for the positive than for the negative direction for the short (*post-hoc p* = 0.04) and medium (*p* < 0.001) but not for the long saccades (*p* = 0.49) (Figure 6B).

## Discussion

We investigated the brain processes related to visual encoding and selection of the next fixation target when observers are scrutinizing natural scenes. We raised the question, whether these processes could be observed in presaccadic electrical brain activity recorded at the scalp. The presaccadic activity was measured in the fixation intervals before saccade onsets while observers were inspecting the first of two scenes, between which change had to be detected. An image heat map was computed for each scene based on individual fixation durations and densities. The temperature differences between the start and end points of saccades on the map were taken as a measure of the expected task-relevance of the information concentrated in specific regions of a scene. Visual encoding was indicated by correctness of change detection. Selection of fixation target was evaluated by saccade directions on the heat maps. We found that both visual encoding and fixation target selection are reflected in presaccadic activity. Visual encoding was associated with presaccadic activity over anterior brain areas for short saccades. Target selection was associated with presaccadic activity over posterior areas for short and medium saccades. Together, we may conclude that presaccadic activity specifies the role of attention in scrutinizing natural scenes.

### Functional significance of the presaccadic activity

What are the factors most likely affecting the amplitude of the presaccadic activity?

The presaccadic interval includes both a shift in covert attention to a novel target and motor preparation for a saccade to that target. Since we did not observe any effects on saccade size (Figure 2B) and the amplitude of the saccadic spike potential (Figure 4B) of our conditions, the oculomotor components of the saccades are equal in strength within each size bin. Therefore the amplitude of the presaccadic activity is likely to reflect a covert shift of attention. An attentional explanation of the presaccadic activity is consistent with previous interpretations of the scalp-recorded presaccadic potentials (Wauschkuhn et al., 1998; Gutteling et al., 2010; Krebs et al., 2012).

Furthermore, the amplitude of the antecedent potential indicates trans-saccadic remapping (Parks and Corballis, 2008). This does not contradict the attentional explanation above since only attended visual features are remapped (reviewed in Mathot and Theeuwes, 2011). In our study the amplitude of the presaccadic activity was larger for saccades in the positive than in the negative direction (Figure 6B). We defined the positive saccade direction, according to fixation heat maps, by higher temperature at the end than start point of a saccade. The temperature difference was higher for saccades in the negative than in the positive direction (Figure 3C), because the *preceding* fixation was shorter for saccades in the positive than in the negative direction (Figure 3A). The fixation *following* was, correspondingly, longer for saccades in the positive than in the negative direction. Since fixation duration indicates amount of attention(Henderson, 2007), the presaccadic activity reflects attention deployed to the *following* fixation location. Thus, the larger amplitude of presaccadic activity for saccades in the positive than in the negative direction may reflect remapping of attended information across a saccade.

It is known that visual information collected during the last 100 ms before a saccade (so called “saccadic dead time”) does not influence the destination of the current saccade (Becker, 1991), but influences the following saccade (Caspi et al., 2004). This suggests functional dissociation of two processes occurring in this interval: accumulation of information for later use and remapping of previously collected information across a saccade to maintain visual stability (Mathot and Theeuwes, 2011). We propose that both processes are reflected in presaccadic activity, taking place in parallel.

Factors of overall alertness or enhanced voluntary processing are less likely to affect selectively the presaccadic activity, because systematic amplitude changes were revealed for saccades of various sizes and directions occurring within the same memorization display, i.e., short enough to render unlikely any systematic changes of alertness or volition.

### Visual encoding

On the fixation heat maps, saccades followed by correct change detection had larger temperature differences (in absolute values) than ones followed by detection failure (Figure 3B). Since temperature difference reflects the informational contrast between two fixation locations, we conclude that successful encoding depends on entering scene regions that are expected to be task-relevant and containing a high concentration of information.

Remarkably, even though the computation of temperature considers fixation durations, these are not sensitive to correctness (Figure 3A). Since temperature also considers fixation density (the spatial distribution of the fixation locations), this must be the variable most directly relevant for correct detection. This conclusion is consistent with previous observations: the number of regional fixations rather than their duration matters for memorization of localized information (Loftus, 1972). Consequently, the heat maps differ for correct detection and failure: in correct detection the information is concentrated in widespread clusters, whereas in failure it is more randomly scattered. Thus, scanning strategy in successful encoding consists of thorough scrutiny of scene regions which expected to be task-relevant.

An effect of correctness on the amplitude of presaccadic activity was found only over anterior brain areas. This effect depended on saccade size and appeared to be associated with short saccades only (Figure 5B). Short saccades mainly occur towards the end of exploring a display (Figure 2A), when potential targets have already been localized and the visual strategy changes to scrutinizing the local regions (Unema et al., 2005; Tatler and Vincent, 2008; Graupner et al., 2011). The amplitude of presaccadic activity was larger in correct detection than in failure (Figure 5A). Since we attributed the presaccadic activity to attention being deployed to the fixation location following, this finding implies that attention is needed for successful encoding. Specifically, successful encoding may depend on scrutiny of the local regions guided by top-down attention rather than global visual exploration of a scene^1^.

### Saccadic target selection

The absolute values of the temperature difference between start and end points of saccade increase with saccade size (Figures 3B,C). Small differences are associated with short saccades because after a small shift in fixation the gaze is likely to land into an image region with similar temperature, whereas after a large shift the gaze may land into a region with a distinct temperature.

The absolute temperature difference was larger for the saccades in the negative than in the positive direction; however, this happened for the medium and long, but not for the short saccades (Figure 3C). Since temperatures depend on fixation duration, this is a consequence of the shorter duration of fixations preceding the medium and long saccades in the positive than in the negative directions (Figure 3A). Temperature indicates how attractive a certain region is as a fixation target. The effect, therefore, may indicate facilitation of fixation target selection if the next fixation target is in an attractive location.

Selection of a saccade target seems to be guided by attractiveness of the next target location only for the medium and long (>30 ms) saccades (Figure 3C). This effect may occur because of the predominant role of bottom-up guidance in long saccades. During initial exploration, visual salience determines the attractiveness of scene regions. The initial exploration is accomplished with long saccades (Figure 2A) (Unema et al., 2005; Pannasch et al., 2008; Graupner et al., 2011). The bottom-up character of the initial exploration offers an explanation for why we did not observe the difference in the presaccadic activity for long saccades (Figure 6B).

Once potential targets have been localized with long saccades, the visual strategy changes to scrutiny of the local regions (Unema et al., 2005; Graupner et al., 2011). This change in strategy is accompanied by a shift from bottom-up to top-down saccade guidance (Findlay and Walker, 1999). The local scanning strategy involves short saccades (Unema et al., 2005; Tatler and Vincent, 2008; Graupner et al., 2011). For the short saccades the informational contrast between current and next target locations is low and, correspondingly, the temperature difference for the positive and negative saccade direction is about equal (Figure 3C). Thus, local scanning is not guided by salient informational content at the next fixation locations. Instead, it may be guided by a top-down mechanism directing the saccades to scene regions which are expected to be task-relevant. This is reflected in the larger presaccadic activity in the positive than in the negative saccade direction over posterior areas (Figure 6B).

Overall, our findings support the notion of different selection mechanisms for short and long saccades, in line with previous observations (Tatler et al., 2006; Foulsham and Kingstone, 2012).

The medium saccades may take up an intermediate position and are guided by combination of bottom-up vs. top-down processes. This is reflected in the difference between positive and negative saccade directions observed in both eye movement and EEG measures (Figures 3C, 6B).

In sum, scalp-recorded electrical brain activity in the presaccadic interval reflects processes related to trans-saccadic perception. We provide evidence for the sensitivity of the presaccadic activity to encoding of visual information and to selection of a target for the next fixation. In these processes the presaccadic activity reflects systematic tendencies in oculomotor behavior which differ in attentional demand.

### Conflict of interest statement

The authors declare that the research was conducted in the absence of any commercial or financial relationships that could be construed as a potential conflict of interest.

## Footnotes

^1^Note, however, that the change blindness phenomenon indicates that attention alone is not sufficient for successful memorization (Mack and Rock, 1998; Rensink, 2002).
